# Role of lingonberry press cake in producing stable herring protein isolates via pH-shift processing: A dose response study

**DOI:** 10.1016/j.fochx.2024.101456

**Published:** 2024-05-11

**Authors:** Jingnan Zhang, Bovie Hong, Mehdi Abdollahi, Haizhou Wu, Ingrid Undeland

**Affiliations:** a Department of Life Sciences- Food and Nutrition Science, Chalmers University of Technology, SE 412 96 Gothenburg, Sweden; b College of Food Science and Technology, Huazhong Agricultural University, Wuhan, Hubei 430070, PR China

**Keywords:** Lipid oxidation, Rancidity, Volatile aldehyde, Natural antioxidant, Berry pomace, Fish by-products, Valorization

## Abstract

The effects of cross-processing lingonberry press cake (LPC) (2.5–30 %, dw/dw) with herring co-products on protein yield, oxidative stability and color of pH-shift-produced protein isolates were investigated. Even at 2.5 % LPC, the formation of volatile oxidation-derived aldehydes, including hexanal, (E)-2-hexenal, heptanal, octanal, and 2,4-heptadienal, were prevented during the actual protein isolate production. Adding 10 % LPC successfully prevented formation of all these aldehydes also during eight days ice storage which was explained by the partitioning of phenolics, especially ideain (1.09 mg/g dw) and procyanidin A1 (65.5 mg/g dw), into isolates. Although higher amounts of LPC (20–30 %) further prolonged the oxidation lag phase, it reduced total protein yield, increased the consumption of acid and base, and darkened protein isolates. Therefore, it is recommended to use 10 % LPC when pH-shift-processing sensitive fish raw materials as a route to mitigate lipid oxidation and at the same time promote industrial symbiosis and more circular food production.

## Highlights

• Adding lingonberry press cake (LPC) improved the process’s lipid removal ability.
• ≥2.5 % LPC limited volatile aldehyde formation during pH-shift-based production.
• ≥10 % LPC prevented volatile aldehyde formation during 8 days of ice storage.
• Total protein yield remained unaffected until the LPC addition ratio reached 10%.
• Lower LPC% saved acid/base usage and rendered lighter color of protein ingredients.

## 1 Introduction

Herring (*Clupea harengus*) is a nutritious fish species rich in *e.g.*, high-quality proteins, long chain (LC) n-3 fatty acids, minerals such as iron, selenium and iodine, as well as vitamins D and B12 (Šližyte et al., 2014, Tuomisto et al., 2020). The consumption of herring has been associated with various advantages for human health, including the enhancement of cardiovascular well-being and improved cognitive function (Tuomisto et al., 2020, De Groot et al., 2012). The global annual herring catch is as high as 1820 thousand tonnes (FAO, 2020), but despite the nutritional advantages, a significant proportion of the landings is dedicated to fodder production; either directly as whole fish or as filleting rest raw materials (Zhang et al., 2024, Luo et al., 2024). The latter make up ∼ 60 % of the fish weight, and results in a major loss of valuable nutrients from the food chain that could have been used directly for human consumption (Wu et al., 2021).

Lingonberry (*Vaccinium vitis-idaea*) is a small, red and tart berry that grows in the boreal forests of the Northern Hemisphere, particularly in Scandinavia (Dróżdż et al., 2017, Viljakainen et al., 2010). In Sweden an estimated 8000 tonnes of wild lingonberry are harvested annually (Casimir et al., 2018). The berries can be consumed raw or cooked, and are commonly used to make lingonberry jam, compote, juice, or syrup (Dróżdż et al., 2017). During lingonberry juice processing, about 20–30 % (w/w) of the fruit is left behind as a press cake (Struck et al., 2016). Although lingonberry press cake (LPC) contains significant amounts of dietary fiber, organic acids, and antioxidants, its integration into food products remains poorly explored. Rather, it is frequently used for purposes like livestock nutrition, biogas generation, or even waste disposal (Bujor et al., 2018, Struck et al., 2016).

The pH-shift method is an effective tool for maximizing the use of fish filleting rest raw materials by extracting valuable proteins (Gehring et al., 2009). This process begins by adjusting the pH of fish-plus-water homogenates to either high or low levels to solubilize the proteins, followed by protein precipitation and separation that occurs at the isoelectric point (pI) of fish proteins (Gehring et al., 2009). However, with heme-rich raw materials, lipid oxidation can occur during the pH-shift-based protein extraction due *e.g.*, to tissue disintegration, exposure to oxygen as well as acidic conditions (Wu et al., 2021). The oxidation of fish lipids leads to the generation of off-flavors, shifts in color appearance, and nutrient degradation, all of which contribute to a decline in product quality and decreased consumer acceptance (Mohammadi et al., 2021).

In earlier research, a new cross-processing concept was developed to tackle the lipid oxidation issue typically seen when pH-shift processing heme-rich fish filleting co-products. This was achieved by blending the fish filleting co-products and 30 % LPC (on a dry weight, dw, basis) before extracting the protein (Zhang et al., 2022). Using this approach, formation of lipid oxidation products such as 4-hydroxy-(E)-2-hexenal (HHE), malondialdehyde (MDA), and 4-hydroxy-(E)-2-nonenal (HNE) was completely prevented throughout the pH-shift protein extraction and the 16-day iced storage of the obtained protein isolates (Zhang et al., 2022). Moreover, it was observed that adding 30 % LPC significantly enhanced the water solubility and gelation capabilities of the resulting protein isolates (Zhang, Ström, et al., 2023).

However, there were also some drawbacks with the new cross-processing concept. First, the total protein yield was reduced even though process modifications were implemented; something which was primarily ascribed reduced protein solubility (Zhang, Ström, et al., 2023). Second, the usage of base solution to reach the targeted protein solubilization pH of 12 increased due to the low pH of the LPC which stems from its abundance of organic acids, *e.g.*, benzoic acid (Klavins et al., 2021). Third, adding 30 % (dw/dw) of LPC to fish co-products dramatically darkened the protein isolates (Zhang et al., 2022). Fourth, based on the life cycle assessment (LCA) results, 30 % LPC-level is suggested to be reduced from the perspective of sustainable production (Coelho et al., 2022, Coelho et al., 2023). Provided the powerful antioxidative ability of LPC compared to other plant-based side streams tested for cross-processing (Zhang et al., 2022), a hypothesis was set up that LPC could be significantly reduced to mitigate the observed challenges, while still preserving the potent antioxidative effects seen both during and after pH-shift processing of herring co-products.

Here, the above hypothesis was tested by progressively reducing the LPC addition level from an initial 30 % (dw/dw) down to a final 2.5 % (dw/dw) during pH-shift processing of herring filleting rest raw materials. To better reflect rancid odour development of the protein isolates, volatile aldehydes were followed as oxidation markers (O’Dwyer et al., 2013) instead of the previously used MDA, HHE and HNE, measured through the 2,4-dinitrophenylhydrazine (DNPH) derivatization combined with liquid chromatography-tandem mass spectrometry (LC-MS/MS) analysis (Abdollahi et al., 2020, Zhang et al., 2022). Five specific volatile aldehydes were selected as markers −hexanal, heptanal, (E)-2-hexenal, 2,4-heptadienal and octanal- which are routinely identified in oxidized fish and other foods rich in either n-3 or n-6 PUFAs (Aitta et al., 2021, Kakko et al., 2022). Effectively applying the innovative cross-processing strategy will pave the way to optimize the extraction of proteins, micronutrients, and phenolic compounds from underutilized herring and lingonberry side streams.

## 2 Materials and methods

### 2.1 Preparation of herring co-products

Fresh co-products (heads and backbones) obtained from industrial herring (*Clupea harengus*) filleting, were provided by Sweden Pelagic AB, located in Ellös, Sweden. Upon their arrival at Chalmers University of Technology in February 2021, the heads and backbones were mixed in equal proportions. A desktop meat grinder with a 4.5 mm perforated plate (C/E22 N; Minerva Omega, Bologna, Italy) was used to grind the mixture. The minced herring co-products were then sealed in plastic bags and preserved at a temperature of −80 °C.

### 2.2 Preparation of lingonberry press cake (LPC)

The press cakes obtained from lingonberry (*Vaccinium vitis-idaea*) juice production were sourced from Grangärde Musteri AB, based in Grangärde, Sweden. They were transported to Chalmers University of Technology in November 2019 after five months of storage at −20 °C. At Chalmers they were stored at −80 °C. The LPC included skins, seeds, leaves, stems, and residual pulp with the leaves quantified to 9 % (ww/ww) and the seeds to 24 %. LPC was ground in a frozen state using a desktop grinder equipped with a 4.5 mm perforated plate (C/E22 N; Minerva Omega, Bologna, Italy) at room temperature. The ground LPC was then stored at −80 °C.

### 2.3 pH-shift processing

**Fig. 1:**
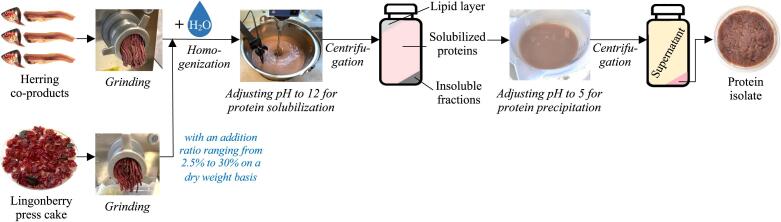
The pH-shift processing of herring co-products with the addition of lingonberry press cake (LPC).

The pH-shift processing of herring co-products with the addition of LPC was conducted following the steps shown in Fig. 1. Initially, both herring co-products and LPC were defrosted in plastic bags under cold tap water until the central temperature reached 0℃. Subsequently, various LPC ratios corresponding to 0, 2.5 %, 5 %, 10 %, 20 % and 30 % of the dry weight of the herring co-products, as presented in the Supplementary Table 1, were mixed with the herring co-products. This combination was further diluted with chilled distilled water at a 1:6 ratio and then uniformly blended using an L5M-A type homogenizer (Silverson, Chesham, UK) operating at 8,000 rpm for 90 s. To achieve protein solubilization, the homogenate (H) was adjusted to a pH of 12.0 using a 2 M NaOH solution, followed by a 15-minute period for protein solubilization. Centrifugation (8,500 × g, 20 min, 4 °C) of the homogenate resulted in three distinct layers: a top lipid layer, an intermediate supernatant, and a bottom sediment. The middle layer, referred to as S1, underwent pH adjustment to 5.0 when LPC was present (Zhang, Ström, et al., 2023) and 5.5 in its absence (Zhang, Ström, et al., 2023), using a 2 M HCl solution. After pH adjustment, another round of centrifugation followed, yielding a second supernatant, termed S2, which was separated from the protein sediment. It is worth noting that the entire pH-shift processing, both with and without LPC, was replicated (*n_e_* = 2) and executed on ice to minimize potential lipid oxidation.

An assessment of protein solubility and protein yield during the two steps of the process, *i.e.*, protein solubilization and protein precipitation, employed repeated sample analyses (triplets, n = 3). The protein concentration in the homogenate and the two supernatants were measured and data were used in equations (1), (2), (3), (4), (5). A derivative of the Lowry protein assay (Markwell et al., 1978) served as the measuring method, capitalizing on the interaction between copper ions and the Folin-Ciocalteu agent. The resulting chromatic response was gauged at a wavelength of 660 nm, with bovine serum albumin (99 %, Sigma-Aldrich Co., Germany) serving as the standard.

(1) $${\mathrm{Solubility}}_{1}\left(\%\right)\;=\frac{c_{\;S1}}{c_{\;H}}\times 100$$

(2) $${\mathrm{Yield}}_{1}\left(\%\right)=\frac{c_{\;S1}\times V_{S1}}{c_{\;H\times }V_{H}}\times 100$$

(3) $${\mathrm{Solubility}}_{2}\left(\%\right)=\frac{c_{\;S2}}{c_{\;S1}}\times 100$$

(4) $${\mathrm{Yield}}_{2}\left(\%\right)=\left(1-\frac{c_{S2}\times V_{S2}}{c_{S1}\times V_{S1}}\right)\times 100$$

(5) $$\mathrm{Totalyield}\left(\%\right)=\frac{c_{S1}\times V_{S1}-c_{S2}\times V_{S2}}{c_{\;H\times }V_{H}}\times 100$$

To prepare protein isolates for ice storage trials, the recovered protein pellet was blended on ice using a stainless-steel spatula. The moisture content was then equalized to 80 % through extra centrifugations (8500 × g, 4 °C). To determine the moisture content between these extra centrifugations, a moisture analyzer (HE73, Mettler Toledo) which heats the samples to 105 °C was used. The protein isolates' pH was eventually set to 7.00 ± 0.05 through the addition of 2 M NaOH. Before subjecting them to ice storage tests, these isolates were stored at −80 °C, with the longest storage time being about two months. While most protein isolate productions were carried out once, those with a 20 % LPC incorporation were done twice.

### 2.4 Storage of protein isolates on ice

The procedure for ice storage of protein isolates was adapted from Wu et al. (2021), with minor alterations. Essentially, to mitigate microbial activity, 200 ppm of streptomycin was integrated into the protein isolates based on their moisture levels. Subsequently, an approximate 27.5 g portion of each protein isolate was spread thinly (around 5–6 mm in depth) into two separate 250 mL Erlenmeyer flasks. Each flask was tightly sealed, encased in aluminum foil to shield from light, and then placed within thermally insulated boxes situated in a 4 °C refrigerated chamber. This storage lasted for a maximum of 16 days. To chemically assess lipid oxidation, periodic samples of roughly 0.7 g were regularly taken. Additionally, a daily qualitative sensorial assessment was performed to detect any onset of rancid odor, measured on a scale ranging from 0 to 100 (see Supplementary Fig. 1 for rancid odor results).

### 2.5 Measurement of volatile aldehydes in protein isolates during ice storage

The analysis of volatile aldehydes, indicative of lipid oxidation levels, was conducted using HS-SPME (Supelco, Bellefonte, PA, USA) in combination with GC–MS (Focus GC ISQ LT; Thermo Fisher Scientific, Austin, TX, USA). The methodology was primarily based on the protocol provided by Sajib et al. (2020), with certain modifications as noted by Zhang et al., 2023, Zhang et al., 2023. In brief, ∼0.7 g of protein isolate sample taken during the ice storage trials were taken from −80 ℃ and immediately thawed under cold running water. After thawing, the samples were weighed to four decimal places and dissolved in 4 ml of Milli-Q water. Using a Potter-Elvehjem piston, the samples were homogenized by pressing against the walls of a glass test tube. The resulting homogenate was then transferred to a 20 ml SPME vial. Afterward, the glass tube and pestle were rinsed with another 4 ml of Milli-Q water, which was added to the initial 4 ml in the vial. As an internal standard, 2.5 mM 3-Methyl-3-buten-1-ol in Milli-Q water was utilized. Two hundred µl of the internal standard was added to the vial, followed by vortexing for 10 s. The volatile compounds were then extracted by stirring at 500 rpm for 20 min at 60℃, after a 5-minute equilibration at the same temperature. Volatiles were adsorbed onto a 75 μm Carboxen/polydimethylsiloxane (CAR/PDMS) coated SPME fiber (Supelco, US). GC–MS analysis involved the desorption of volatiles at 300℃ for 10 min, followed by separation on a fused silica ZB-1701 capillary column (Phenomenex, 30 m x 0.32 mm, 1 μm). The MS operated in electron ionization mode, scanning ions from 10 to 250 amu. Markers for lipid oxidation, hexanal, (E)-2-hexanal, heptanal, octanal, and 2,4-heptadienal, were selected due to their abundance in the volatiles detected (Zhang, 2023) and their prevalence in oxidized fish and other samples containing high amounts of n-3 or n-6 PUFA (Aitta et al., 2021, Kakko et al., 2022). Specific details on the retention time and SIM mass linked to these volatile aldehydes can be found in Supplementary Table 2.

### 2.6 Proximate composition analysis of raw materials and protein isolates

Moisture and ash contents were measured by subjecting the samples to temperatures of 105℃ and 550℃, respectively (AOAC, 2005). Crude protein content was quantified using the Dumas method with a LECO nitrogen analyzer (TruMac-N; LECO, St. Joseph, MI, USA) as detailed by Marcó et al. (2002). Nitrogen-to-protein conversion ratios of 5.58 were applied for herring co-products and isolates, and 5.4 for LPC (Mariotti et al., 2008, Zhang et al., 2022). Crude lipid content was determined by using a modified version of Lee's method involving chloroform and methanol extraction processes (Zhang et al., 2022).

### 2.7 Hemoglobin (Hb) measurements of herring co-products

Frozen co-products of herring were combined with liquid nitrogen and subsequently milled into a powder using a chopper. The quantification of Hb was executed using the Hornsey acid hematin method (Hornsey, 1956).

### 2.8 Total phenolic content (TPC) measurements of LPC and protein isolates

The phenolic compounds were extracted following the procedure detailed in Zhang et al. (2022), employing a 70 % methanol solution containing 1 % trifluoroacetic acid as the extraction solvent. TPC was quantified utilizing a modified version of the Folin-Ciocalteu colorimetric assay (Oliveira et al., 2019), and the TPC values were expressed as gallic acid equivalents (GAE) per 100 g of the sample on a dry weight (dw) basis.

### 2.9 Analysis of key phenolic compounds in LPC and protein isolates

The extraction of phenolics was conducted according to Oliveira et al. (2019) with modifications. In brief, 0.2 g of freeze-dried sample was mixed with 3 mL of 70 % acidified methanol (0.3 % HCl) in a 50 mL centrifugation tube, followed by 30 s of vortexing. Subsequently, the tubes were placed in an ultrasonication bath for 15 min at room temperature, with intermittent shaking every 5 min. Afterwards, centrifugation at 5,000 × g for 10 min separated the supernatant. The extraction procedures were repeated twice, and the supernatants containing the extracted phenolics were collected. The supernatants were then centrifuged at 3,000 × g for 10 min to remove the insoluble fractions. The supernatants were stored at −80 °C until analysis. Phenolics quantification was performed using a liquid chromatography-tandem mass spectrometry (LC-MS/MS) system comprising a 6500 + QTRAP triple-quadrupole mass spectrometer (AB Sciex, Stockholm, Sweden) with an ESI Turbo Spray Ion Drive source, operated in positive-ion mode. Chromatographic separations utilized a Premier BEH C18 1.7 μm 2.1x50mm column (Waters, Solna, Sweden). The mobile phases were LC-MS grade water (solvent A) and MeOH (solvent B), both with 0.1 % formic acid, used in a gradient elution as follows: 0–2 min, 2 % B; 2–2.5 min, 10 % B; 2.5–4 min, 25 % B; 4–4.5 min, 45 % B; 4.5 min, 100 % B (held for 30 *sec*), and 4.5–6 min, 2 % B. The flow rate was set to 0.8 mL/min, column temperature was maintained at 50 °C, and the autosampler temperature was 12 °C. Multiple reaction monitoring (MRM) transitions for each analyte were optimized via direct infusion at a concentration of 25 mM for each phenolic molecule. Two Q1/Q3 pairs were determined for each analyte; the more sensitive of the two was used for quantification, while the other served as a qualifier for compound identity verification. The retention time window for the scheduled MRM was 1 min for each analyte. Based on previous studies of LPC subjected to pH-shift processing (Zhang et al., 2022, Lei et al., submitted for publication), in the present study, cyanidin 3-O-galactoside (Cy3Gal, also known as 'ideain') and procyanidin A1 were used as standards, and the data are expressed as μg/g dw, or mg/g dw.

### 2.10 Color measurements of protein isolates

A colorimeter (CR-400; Konica Minolta, Osaka, Japan) was used to assess the color characteristics of protein isolates in the CIE *L^∗^a^∗^b^∗^* color space, following the guidelines set by Zhang et al. (2022). The influence of varying LPC addition on the color characteristics of the herring protein isolates was determined via the *△E_2000_*, derived from the CIEDE2000 color deviation equation based on primary color values (Sharma et al., 2005).

### 2.11 Statistical analysis

To identify significant differences among samples and comprehend the time-based changes in volatile aldehyde formation for a specific sample at stipulated intervals, a statistical analysis was conducted using the SPSS Statistics software (version 27, IBM, NY, USA). The process included a one-way analysis of variance (ANOVA) followed by Duncan's post-hoc analysis. A significance level (p) was set at 0.05, below which the differences were considered significant.

## 3 Results and discussion

### 3.1 Lipid oxidation in protein isolates produced with different ratios of LPC

The oxidation of unsaturated fatty acids produces peroxyl radicals, which subsequently react with other unsaturated fatty acids to form hydroperoxide intermediates. Further chemical reactions such as fragmentation and rearrangement lead to the formation of aldehydes, some of which are stable over time and can be reliably quantified using *e.g.*, GC (Schneider, 2009, Kakko et al., 2022, Kakko et al., 2022). In this study, hexanal, (E)-2-hexenal, heptanal, octanal, and 2,4-heptadienal were quantified. The selection of these volatile aldehydes as markers of lipid oxidation was based on a preliminary study that explored the volatile profiles of fresh and oxidized herring protein isolates produced by the pH-shift method (Zhang, 2023). These volatile aldehydes are also reported to be abundant or used as lipid oxidation markers in herring oils (Aitta et al., 2021), herring protein isolates (Kakko, Aitta, et al., 2022), silver carp skin, belly, and mince (Kunyaboon et al., 2021), and large yellow croaker meat (Zhao et al., 2021). Hexanal is a six-carbon saturated aldehyde which is primarily linked to n-6 PUFAs oxidation (Jiarpinijnun et al., 2022). It can also be produced from the breakdown of other preformed volatiles, such as 2-octenal and 2,4-decadienal (Sajib & Undeland, 2020). Another six-carbon aldehyde, (E)-2-hexenal, is formed during linolenic acid oxidation (Aitta et al., 2021, Kakko et al., 2022). Heptanal, a seven- carbon aldehyde, and octanal, an eight-carbon aldehyde, can be generated from several unsaturated fatty acids, including, but not limited to, palmitoleic acid, linoleic acid and eicosadienoic acid (Sutaria et al., 2022). The compound 2,4-heptadienal is a seven-carbon aldehyde that can be formed when eicosapentaenoic acid (EPA) undergoes oxidation (Lee et al., 2003).

**Fig. 2:**
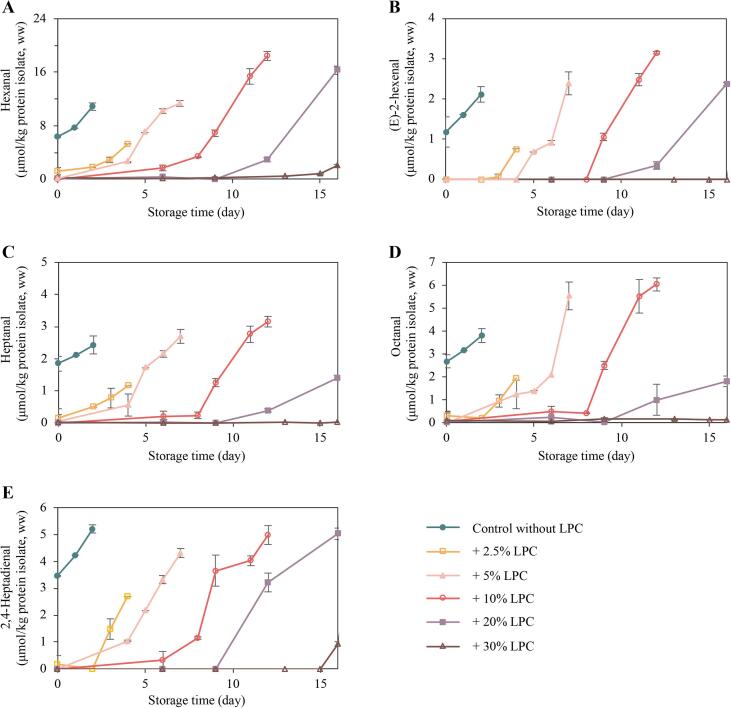
Formation of lipid oxidation-derived volatile aldehydes, including hexanal (A), (E)-2-hexenal (B), heptanal (C), octanal (D), and 2,4-heptadienal (E), during ice storage of protein isolates produced without and with lingonberry press cake (LPC) addition (2.5–30 %, on a dry weight basis). Data are presented as mean values ± standard deviation (n ≥ 2).

As depicted in Fig. 2, elevated levels of all marker aldehydes were detected in the freshly prepared control samples at Day 0, and the levels continued to increase until Day 3, on which day the sampling stopped due to an intense rancid odor. These findings were consistent with previous research indicating elevated concentrations of lipid hydroperoxides, MDA, HHE, HNE, and total carbonyls in the herring protein isolates produced by the pH-shift method (Abdollahi et al., 2020, Zhang et al., 2023). The observed lipid oxidation can be attributed to *e.g.*, the high Hb content of herring co-products −in the present study 972.76 ± 5.09 μmol/kg wet weight (ww). Hb can decompose lipid hydroperoxides into free radicals and/or oxidize to ferryl heme iron and protein based free radicals, both pathways stimulating lipid oxidation (Wu et al., 2021, Zhang et al., 2024). Earlier research has revealed how the protein precipitation step at pH 5.5 can stimulate Hb-deoxygenation and metHb formation, which ultimately stimulate hemin loss and thereby hydroperoxide breakdown into radicals (Kristinsson and Hultin, 2004, Undeland et al., 2004). Additionally, co-precipitation of Hb with myofibrils and/or membranes at pH 5.5 can bring Hb and hemin close to the phospholipid oxidation substrate (Wu et al., 2021). Specific pH-shift process steps, particularly water addition and the first centrifugation, can dilute ascorbic acid and sediment α-tocopherol, respectively, hereby disturbing the endogenous anti- to pro-oxidant balance (Wu et al., 2021). Further to this, the high-speed homogenization step and the unfolding/refolding of proteins can disrupt the fish muscle microstructure and expose membranal phospholipids to pro-oxidants like Hb (Kristinsson and Hultin, 2004, Wu et al., 2021).

As shown in Fig. 2, even at the lowest LPC addition (2.5 %, dw/dw), no significant change (p > 0.05) in the volatile aldehyde contents was detected in the freshly made protein isolates., *i.e.*, on Day 0. This finding indicated a powerful antioxidant capacity of LPC. Berries are recognized for their high concentration of antioxidants, *e.g.*, polyphenols such as flavonoids, anthocyanins, and phenolic acids (Bujor et al., 2018, Dróżdż et al., 2017, Mikulic-Petkovsek et al., 2012, Tulio et al., 2008, Yang et al., 2019). Among different berries, lingonberry has been noted for its high levels of proanthocyanidins, anthocyanins, and quercetin, as well as phenolic acids including benzoic acid, ellagic acid, and gallic acid (Bujor et al., 2018, Dróżdż et al., 2017). Lingonberry fruit extracts, prepared with 60:40 ethanol to water, displayed notable 1,1-diphenyl-2-picryl-hydrazyl (DPPH) radical scavenging activity, while extracts using ethyl acetate exhibited effective cupric reducing antioxidant capacity (CUPRAC) (Dróżdż et al., 2017). In addition to the fruits, the leaves and stems of lingonberry have been reported to be significant sources of natural antioxidants illustrated as an impressive DPPH radical scavenging activity (Bujor et al., 2018). The LPC used in the present study mainly consists of skins, seeds, and residual pulp. Its TPC content was determined as 3.60 ± 0.09 g gallic acid equivalents (GAE)/100 g, dry weight (dw). It is important to stress that during the pH-shift processing, the LPC is first extracted with water at alkaline pH and the extract is then subjected to a pH representing the pI of muscle proteins, ∼pH 5.5. This indicates that the alkali-soluble molecules, which remain soluble at pH 5.5 or may co-precipitate with the protein isolate, are expected to be key antioxidants. To investigate the solubility pattern of LPC antioxidants during pH-shift processing, LPC alone was subjected to pH-shift processing in a recent study (Lei et al., submitted for publication). The different LPC fractions obtained during the processing, *i.e.*, pellets and supernatants from the first and second centrifugations, were blended with Hb-fortified washed cod mince (WCM) to study their antioxidant capacity during ice storage. The fractions that exhibited the best antioxidant abilities were further separated according to polarity and subjected to analysis of phenolics. Cyanidin 3-O-galactoside (Cy3Gal), also known as 'ideain,' and procyanidin A1 were identified as the two key compounds (Lei et al., submitted for publication). In the current study, the Cy3Gal and procyanidin A1 content of the LPC were determined as 58.50 ± 0.73 mg/g, dw and 4.22 ± 0.03 mg/g, dw, respectively.

During the 16 days of ice storage of protein isolates, it was shown that the increased LPC addition ratio delayed the formation of volatile aldehydes (Fig. 2), which aligned with the hypothesis. As an example, the content of hexanal did not significantly increase until Day 4 with 2.5 % LPC, until Day 5 with 5 % LPC, until Day 9 with 10 % LPC, and until Day 12 with 20 % LPC. With 30 % LPC, aldehyde formation was completely prevented throughout the 16 days of ice storage. The results of (E)-2-hexenal, heptanal, octanal, and 2,4-heptadienal corroborated the conclusions made based on the hexanal data, as shown in Fig. 2. The same was true for rancid odor data collected by internal sensory analysis during the storage (Supplementary Fig. 1). The reason for this clear dose–response behavior was strongly related to the TPC of the protein isolates. As the addition of LPC increased from 2.5 % to 30 % (dw/dw), the TPC of isolates went from 0.27 ± 0.02, 0.34 ± 0.02, 0.43 ± 0.01, 0.67 ± 0.03, to 0.91 ± 0.03 g GAE/100 g dw. The finding that 30 % LPC entirely inhibited the formation of all five volatile aldehyde markers during 16 days of ice storage aligns with previous study revealing that 30 % LPC completely prevented the production of MDA, HHE, and HNE in protein isolates derived from herring and salmon co-products over 16-days on ice (Zhang et al., 2022). This finding does not only indicate robustness of LPC antioxidant activity across studies, but also reveals good agreement between the two approaches for evaluating lipid oxidation. Similarly, Damerau et al. (2020) reported that adding adding 3 % (w/w) dried industrial lingonberry press cake to minced, deskinned herring fillets significantly reduced the formation of volatile oxidation products over 10 months of frozen storage (Damerau et al., 2020).

The two key compounds defined in LPC, Cy3Gal and procyanidin A1 (Lei et al., submitted for publication), were also measured in the cross-processed protein isolates. As shown in Table 1, when increasing the LPC addition ratio from 2.5 % to 30 % (dw/dw), the concentration of Cy3Gal gradually increased from 0.08 ± 0.00 mg/g dw to 2.22 ± 0.01 mg/g dw, and procyanidin A1 from 4.62 ± 0.50 μg/g dw to 174.66 ± 1.52 μg/g dw. Based on the levels of Cy3Gal and procyanidin A1 in LPC and final protein isolates, it was concluded that 5 %, 14 %, 21 %, 16 %, and 16 % of the Cy3Gal as well as 4 %, 9 %, 17 %, 15 % and 18 % of the procyanidin A1 were transferred from LPC to isolates at the different tested LPC-levels 2.5 to 30 %. Given the fact that the LPC constituted 0.3 %, 0.7 %, 1.3 %, 2.4 % and 3.3 % wet weight % of the total fish + water + LPC system at start of the pH-shift process, it can be seen that the two flavonoids were concentrated throughout the process. Further investigations shall investigate whether pre-treating the LPC can enhance the retention rates of key phenolic compounds in the protein isolates.

**Table 1:** Composition of herring co-products, lingonberry press cake (LPC), and protein isolates: cyanidin 3-o-galactoside (Cy3Gal) content, procyanidin A1 content, and proximate composition including crude protein, crude lipid, and ash content. Data are presented as mean values ± standard deviation (n ≥ 2) on a dry weight (dw) basis.

	LPC addition (%, dw/dw)	Cy3Gal (mg/g dw)	Procyanidin A1 (μg/g dw)	Protein (% dw)	Lipids (% dw)	Ash (% dw)
Herring co-products		/	/	54.2 ± 2.9^c^	30.3 ± 0.7^a^	13.5 ± 1.4^a^
LPC		58.50 ± 0.73	4221.43 ± 28.71	6.9 ± 0.3^d^	17.7 ± 1.2^b^	2.5 ± 0.1^b^
Protein isolates	0	/	/	82.0 ± 0.3^a^	13.7 ± 0.3^c^	3.6 ± 0.2^b^
	2.5	0.08 ± 0.00^e^	4.62 ± 0.50^e^	81.5 ± 3.6^a^	13.7 ± 0.2^c^	3.3 ± 0.0^b^
	5	0.39 ± 0.02^d^	18.03 ± 0.42^d^	79.8 ± 1.3^a^	13.7 ± 0.3^c^	3.2 ± 0.1^b^
	10	1.09 ± 0.03^c^	65.50 ± 0.87^c^	78.1 ± 1.5^a^	13.4 ± 0.5 ^cd^	3.2 ± 0.1^b^
	20	1.58 ± 0.06^b^	108.84 ± 1.15^b^	73.7 ± 0.6^b^	12.8 ± 0.3^d^	3.5 ± 0.3^b^
	30	2.22 ± 0.01^a^	174.66 ± 1.52^a^	70.2 ± 0.8^b^	12.4 ± 0.1^e^	3.5 ± 0.1^b^

Data within the same column carrying a different superscript letter are significantly different (p < 0.05).

### 3.2 Protein solubility and protein yield during processing

**Fig. 3:**
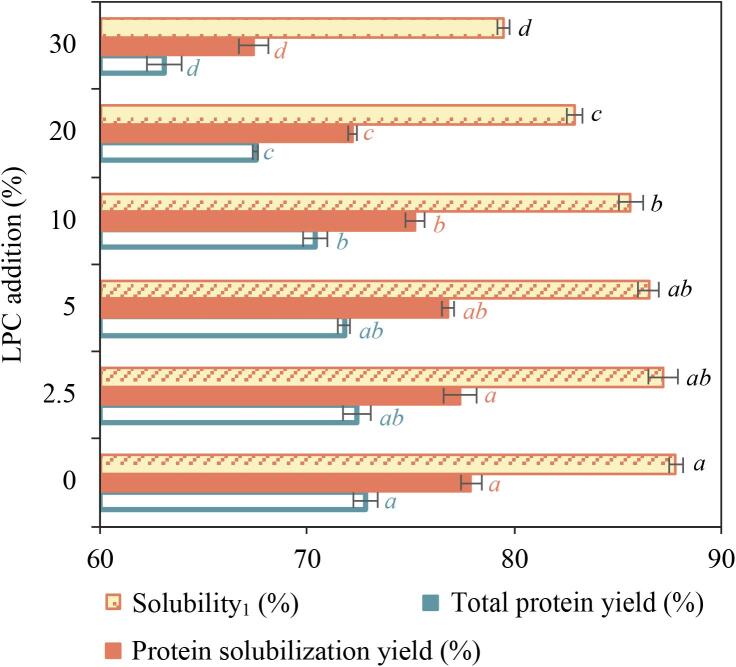
Protein solubility (*Solubility_1_*) and protein solubility yield in the first step of the pH-shift process, and total protein yield during the entire pH-shift processing of herring co-products without and with lingonberry press cake (LPC) addition (2.5–30 %, on a dry weight basis). Data are presented as mean values ± standard deviation from two experiments (*n_e_* = 2) and three samplings (n = 3) from each process. Significant differences (p < 0.05) are denoted by different lowercase letters within each data group.

When extracting proteins from herring co-products using the pH-shift method, both protein solubility and protein yield were observed over the two separation steps, with or without the inclusion of 2.5–30 % LPC (dw/dw). The addition ratio of LPC showed no influence on protein solubility and protein yield during the protein precipitation step, consistently registering at about 7 % and 94 %, respectively (see Supplementary Table 3). However, the protein solubilization step experienced notable changes, which aligned with the hypothesis. As the proportion of LPC increased, there was a discernible decline in both protein solubilization and its resultant solubilization yield, leading to a reduction in the overall yield (Fig. 3). At 30 % LPC addition, solubility dropped from 88 % to 80 %, protein solubilization yield from 78 % to 67 % and total yield from 73 % to 63 % compared to the herring control without LPC (Fig. 3).

These results align with Zhang et al., 2022, Zhang et al., 2023, Zhang et al., 2023 where the reduction was explained by polyphenol-induced precipitation of herring proteins preventing their solubilization at alkaline pH. When exposed to an alkaline pH, phenols may be oxidized to quinone derivatives, which can then react with muscle proteins, particularly myofibrillar proteins, to form covalent bonds, ultimately inducing cross-linked protein polymers (Guo & Xiong, 2021). Myosin, being the most abundant and functional component in myofibrillar proteins, was earlier reported to become cross-linked when interacting with phytophenols (Choi & Kim, 2009). In the present study, the co-precipitates formed during the protein solubilization step were lost in the first centrifugation step as they partition into the sediment (Gehring et al., 2009). As expected, reducing LPC addition from 30 % (dw/dw) counteracted the loss of protein solubility at pH 12, and therefore enhanced the protein yields. Compared to the 30 % LPC addition, decreasing the LPC addition to 20 % resulted in an increase in total protein yield by 5 %, and decreasing the LPC addition to 10 % led to an increase in total protein yield by 13 %. Meanwhile, incorporating LPC-levels as low as 5 % and 2.5 % did not significantly influence protein solubility or protein yield when compared with the control sample without LPC (Fig. 3).

### 3.3 Required amounts of alkali and acid during processing with different ratios of LPC

**Fig. 4:**
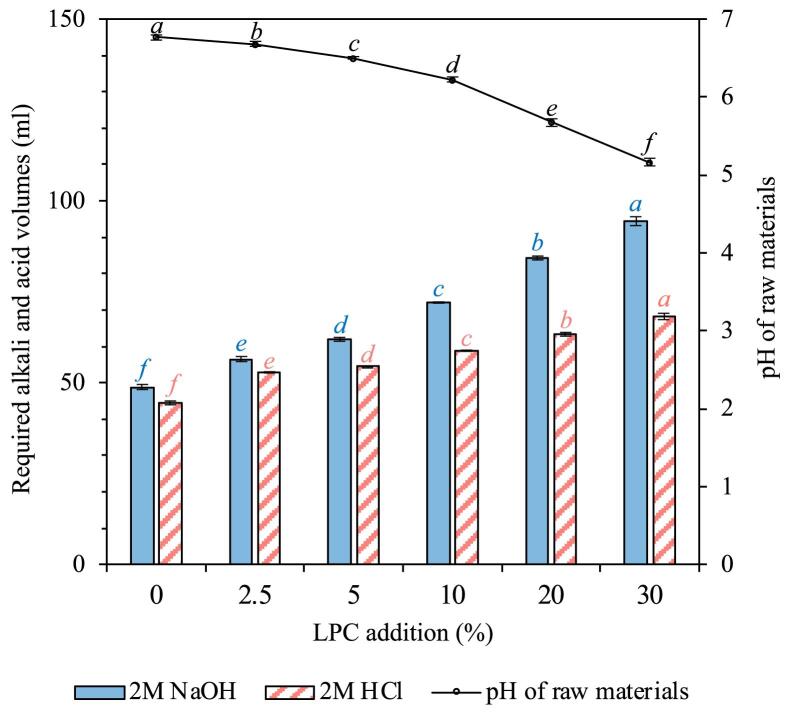
Effect of lingonberry press cake (LPC) addition on the pH of raw materials and the required volumes of alkali (2 M NaOH) and acid (2 M HCl) during pH-shift processing of 500 g of herring co-products with different LPC addition ratios (lines). Data are presented as mean values ± standard deviation (*n_e_* = 3). Significant differences (p < 0.05) are denoted by different lowercase letters within each data group.

As shown by the black line in Fig. 4, when adding 2.5–30 % (dw/dw) of LPC, the pH of the raw material mixture subjected to the pH-shift process continuously decreased from 6.77 ± 0.03 for the control sample, to 5.16 ± 0.05 at 30 % LPC addition (p < 0.05). This drop can be linked to the intrinsic acidic nature of LPC, which has a pH of 3.05. This acidity is due to its constituent organic acids, including benzoic, citric, malic, cinnamic, tartaric, fumaric, and shikimic acids as reported in previous studies (Klavins et al., 2021, Mikulic-Petkovsek et al., 2012, Viljakainen et al., 2010). This agrees with a past investigation where a 30 % addition of LPC, which in itself has a pH of 2.9, to herring or salmon filleting co-products pH decreased their pH from 6.9 to 5.0 for herring and from 6.2 to 4.7 for salmon (Abdollahi et al., 2020). Due to this decline in initial raw material pH, as hypothesized, the usage of alkali (2 M NaOH) was significantly increased. As depicted by the blue bars in Fig. 4, a 30 % LPC addition amplified the requirement of 2 M NaOH from 48.8 ± 0.6 ml to 94.4 ± 1.3 ml for every 500 g of herring co-products, in comparison to the LPC-free sample. Gradually reducing the LPC addition from 30 % to 20 %, 10 %, 5 % and 2.5 % saved the consumption of 2 M NaOH by 11 %, 24 %, 34 % and 40 %, respectively. Accordingly, when using 30 % LPC, the amount of 2 M HCl needed for protein precipitation was dramatically increased by 53 % compared to the control. However, by reducing the LPC addition to 20 %, 10 %, 5 % and 2.5 %, the need for acid was then decreased by 7 %, 14 %, 20 % and 23 %, respectively (Fig. 4 (red bars)). Thus, from a process economic and environmental perspective, lower levels of LPC are advantageous.

### 3.4 Protein, lipid and ash content

This research reaffirmed the effectiveness of the pH-shift method in concentrating proteins, as reported for bigeye snapper head by-products (Panpipat & Chaijan, 2017), common carp muscle (Singh et al., 2019) and tilapia frame by-products (Chomnawang & Yongsawatdigul, 2013), irrespective of LPC inclusion. This is a result of the methodś efficacy in removing lipids and ash, which accumulate in the lipid layer at the top and in the sediment, respectively, after the first centrifugation step (Gehring et al., 2009). The separation of lipids, solubilized proteins, and bones is enabled by their differing densities, in line with previous research on the pH-shift method (Gehring et al., 2009). In alignment with the hypothesis, the protein isolate composition was impacted when LPC exceeded 20 % (dw/dw) (Table 1). First, at 20 % and 30 % LPC, the protein content was significantly reduced from 82 to 74 and 70 g/100 g dw, respectively, compared to the control sample. This was predictable as LPC contains only 7 g of protein per 100 g dw while it has a high carbohydrate level. Second, relative to the control sample, a 20 % inclusion of LPC marginally but significantly reduced the lipid content in protein isolates from 13.7 to 12.8 g/100 g dw, which further declined to 12.4 g/100 g dw at a 30 % LPC addition (Table 1). This observation suggests that LPC enhances the ability to remove lipids during the processing. When the pH-shift method is applied to fish co-products, the neutral lipids are mostly removed from the solubilized proteins as a floating lipid layer while phospholipids can be sedimented; both events generated by polarity and density differences (Zhang et al., 2023, Zhang et al., 2023). Nevertheless, the lipid removal capability of the pH-shift method can be influenced by the emulsification ability of proteins and saccharides in the system (Vareltzis & Undeland, 2008). The observed lipid reduction can potentially be attributed to the increased polarity of the system caused by phenolic compounds and by the strong emulsification ability of LPC polysaccharides. The ash content of the cross-processed protein isolates was statistically independent of LPC addition (p > 0.05), which was unexpected since more NaOH and HCl were used along with more LPC (see Fig. 3); this should theoretically form more NaCl. The present result could be explained by a counteracting effect from a gradual dilution of the herring bone-derived ash by LPC (Zhang, Abdollahi, et al., 2023).

### 3.5 Color of protein isolates

**Fig. 5:**
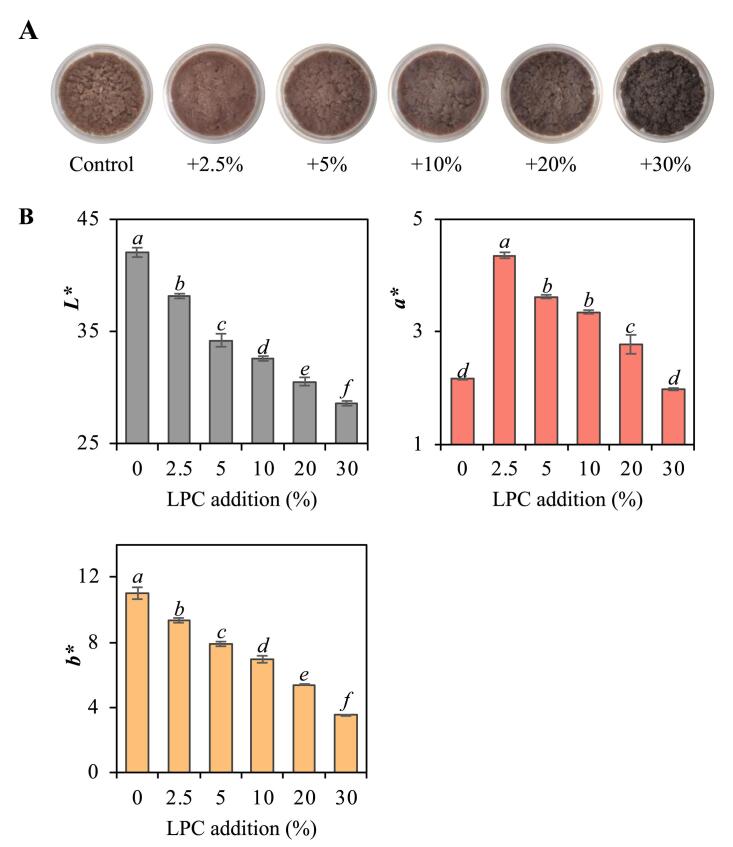
Visual appearance (A) and color attributes using the L*a*b* color space (B) of protein isolates made with increasing addition of lingonberry press cake (LPC). Data are presented as mean values ± standard deviation (n ≥ 3). Significant differences (p < 0.05) within each dataset (L*-, a*- and b*-values) are indicated by different letters.

Fig. 5A illustrates the influence of the LPC addition ratio on the color of the protein isolates. Compared to the control sample, increasing the LPC addition ratio from 2.5 % to 30 % (dw/dw) led to a continuous increase in the color change, as indicated by the *△E_2000_* values of 4.7, 7.5, 8.8, 10.6 and 12.5, respectively. These results supported the hypothesis that decreasing the LPC addition could minimize color changes. The protein isolates visually turned darker with increasing LPC addition (Fig. 5A), which was confirmed by significantly (p < 0.05) decreased L*-values (Fig. 5B). This color change could be related to the pH sensitivity of the anthocyanins in LPC. It is recognized that these compounds transition from a bright red shade at acidic levels to a darker purple at neutral to higher pH due to the reversible chalcone-flavanone conversion (Fossen et al., 1998). Interestingly, there was a significant increase in a*-values at 2.5 % LPC compared to the control, but at further increased LPC levels, the a*-values decreased. These observations were likely attributed to the antioxidative properties of LPC, which may inhibit the conversion of oxy-/deoxy-Hb and −myoglobin (Mb) into the brown metHb or metMb during the protein precipitation step at pH 5 (Wu et al., 2021). However, as LPC levels were raised, the purple-blue colour of the anthocyanins gradually overshadowed the red colour of oxy-/deoxy-Hb/-Mb. Similar logic most likely explains the gradual decrease in b*-values (yellow-blue axis). When going from 0 to 30 % LPC, there was a stepwise masking of the inherent yellowness of the protein isolate by purple-blue pigments.

## 4 Conclusions

This study rigorously evaluated the impact of varying lingonberry press cake (LPC) ratios (2.5–30 %, dw/dw) on protein isolation from herring co-products using the pH-shift method. The results clearly demonstrate that inclusion levels as low as 2.5 % LPC effectively prevent lipid oxidation in freshly made protein isolates, with no significant increase of key aldehydes such as hexanal and heptanal, which were present in the control samples. Higher LPC-levels were needed to delay oxidation also during protein isolate storage on ice, and balancing stabilization efficacy, color, and acid and base consumption, a concentration of 10 % LPC emerged as optimal. At this inclusion ratio, aldehyde formation was significantly delayed for at least eight days on ice without reducing protein yield, which was observed with higher LPC ratios. The 10 % inclusion rate also afforded reductions in usage of chemicals—up to 40 % for NaOH and 23 % for HCl—thereby enhancing the sustainability of the process. Phenolic compound analysis corroborated the antioxidant capacity of LPC, showing a direct correlation between LPC concentration and the levels of cyanidin 3-O-galactoside and procyanidin A1 in protein isolates. At the suggested LPC-level of 10 %, these anthocyanins were present in protein isolates at 1.09 ± 0.03 mg/g dw, and 65.50 ± 0.87 μg/g dw, respectively.

This study underscores the potential for jointly integrating marine and plant-based rest raw materials in food processing, which could facilitate more sustainable and economically advantageous practices in the industry. This approach not only stabilizes protein extracts but also aligns with clean-label standards, which are increasingly demanded by consumers. While promising, these results are limited to laboratory-scale experiments. Scale-up processes may introduce variables that could affect the efficiency and outcomes observed here. Alongside deeper mechanistic understanding on the antioxidant action, future research should thus focus on pilot-scale studies to validate these findings under conditions closer to industrial ones. Further investigation into the environmental impacts of integrating different rest raw materials at larger scales would also be beneficial, paving the way for a broader application of industrial symbiosis in food production.

## CRediT authorship contribution statement

**Jingnan Zhang:** Conceptualization, Formal analysis, Investigation, Methodology, Supervision, Visualization, Writing – original draft, Writing – review & editing. **Bovie Hong:** Formal analysis, Investigation. **Mehdi Abdollahi:** Resources. **Haizhou Wu:** Methodology, Writing – review & editing. **Ingrid Undeland:** Conceptualization, Funding acquisition, Methodology, Writing – review & editing.

## Declaration of competing interest

The authors declare that they have no known competing financial interests or personal relationships that could have appeared to influence the work reported in this paper.

## Data Availability

Data will be made available on request.
