# Supplementary material for: Role of lingonberry press cake in producing stable herring protein isolates via pH-shift processing: A dose response study
Source: Food Chem X. 2024 May 11;22:101456. doi: 10.1016/j.fochx.2024.101456 (PMC11130683; doi:10.1016/j.fochx.2024.101456)
Supplement: Supplementary Data 1 [file mmc1.docx]

***Supplementary Materials*** *for*

**Role of Lingonberry Press Cake in Producing Stable Herring Protein Isolates via pH-Shift Processing: A Dose Response Study**

Jingnan ZHANG^a^*, Bovie HONG^a^, Mehdi ABDOLLAHI^a^, Haizhou WU^a,b^, Ingrid UNDELAND^a^

^a^ Department of Life Sciences- Food and Nutrition Science, Chalmers University of Technology, SE 412 96 Gothenburg, Sweden

^b^ College of Food Science and Technology, Huazhong Agricultural University, Wuhan, Hubei 430070, PR China

**Supplementary Table 1**. The amount of herring co-products and lingonberry press cake (LPC) used for pH-shift processing; LPC was added in an amount that corresponded to 2.5%-30% of herring co-products’ dry weight (dw); distilled water was added in an amount which corresponded to 6 times of the total raw materials’ wet weight.

| LPC addition  (%, dw/dw) | Combination of raw materials and water (g) | | |
| --- | --- | --- | --- |
|  | Herring co-products | LPC | Water |
| 0 | 500 | 0 | 3000.0 |
| 2.5 | 500 | 10.4 | 3062.1 |
| 5 | 500 | 25.9 | 3155.3 |
| 10 | 500 | 51.8 | 3310.5 |
| 20 | 500 | 103.5 | 3621.1 |
| 30 | 500 | 155.3 | 3931.6 |

**Supplementary Table 2.** Retention time (RT) and SIM ion mass for identification of volatile aldehydes used as the indicators of lipid oxidation. For SIM mass, the number in bold indicates the target ion mass and the rest are the reference ions.

| Compound name | RT (min) | SIM mass |
| --- | --- | --- |
| Hexanal | 13.28 | 44 + 56 |
| (E)-2-hexenal | 17.60 | 40 + 55 + 83 |
| Heptanal | 18.82 | 44 + 70 + 81 |
| Octanal | 21.97 | 43 + 56 + 84 |
| 2,4-Heptadienal | 23.10 | 40 + 53 + 81 + 110 |

**Supplementary table 3**. Protein solubility and yield measured during the protein precipitation step when applying pH-shift processing to herring co-products wo/w adding LPC at addition ratios from 2.5 to 30% (dw/dw). Data are given as percentages showing mean values ± standard deviation (*n_e_*=2, n=3).

| LPC addition (%) | Protein precipitation step | |
| --- | --- | --- |
|  | Protein solubility (%) | Protein precipitation yield (%) |
| 0 | 7.4 ± 0.2^a^ | 93.5 ± 0.1^a^ |
| 2.5 | 7.3 ± 0.0^a^ | 93.5 ± 0.0^a^ |
| 5 | 7.2 ± 0.0^a^ | 93.6 ± 0.1^a^ |
| 10 | 7.2 ± 0.1^a^ | 93.6 ± 0.1^a^ |
| 20 | 7.3 ± 0.1^a^ | 93.5 ± 0.1^a^ |
| 30 | 7.3 ± 0.2^a^ | 93.6 ± 0.2^a^ |

Different small letters in each column show a significant difference (p<0.05).

**Supplementary Figure 1**. Rancid odor detected during ice storage of protein isolates. Isolates were stored in 250 mL E-flasks and the headspace above the samples was smelled as previously described (Sannaveerappa et al., 2007).

***Reference***

Sannaveerappa, T., Carlsson, N. G., Sandberg, A. S., & Undeland, I. (2007). Antioxidative properties of press juice from herring (*Clupea harengus*) against hemoglobin (Hb) mediated oxidation of washed cod mince. *Journal of Agricultural and Food Chemistry*, *55*(23), 9581–9591. https://doi.org/10.1021/jf071237i
